# Health-Related Quality of Life Measured by EQ-5D in Relation to Hospital Stay and Readmission in Elderly Patients Hospitalized for Acute Illness

**DOI:** 10.3390/ijerph17155333

**Published:** 2020-07-24

**Authors:** Cheng-Fu Lin, Yu-Hui Huang, Li-Ying Ju, Shuo-Chun Weng, Yu-Shan Lee, Yin-Yi Chou, Chu-Sheng Lin, Shih-Yi Lin

**Affiliations:** 1 Center for Geriatrics & Gerontology, Taichung Veterans General Hospital, Taichung 40705, Taiwan; chengfue@gmail.com (C.-F.L.); hyhui@vghtc.gov.tw (Y.-H.H.); j01i02m3@yahoo.com.tw (S.-C.W.); yslee@vghtc.gov.tw (Y.-S.L.); yinyichou@gmail.com (Y.-Y.C.); billlin@vghtc.gov.tw (C.-S.L.); 2 Division of Occupational Medicine, Department of Emergency, Taichung Veterans General Hospital, Taichung 40705, Taiwan; 3 Department of Nursing, Taichung Veterans General Hospital, Taichung 40705, Taiwan; yuiju@vghtc.gov.tw; 4 School of Nursing, Chung Shan Medical University, Taichung 40201, Taiwan; 5 Division of Nephrology, Department of Internal Medicine, Taichung Veterans General Hospital, Taichung 40705, Taiwan; 6 Institute of Clinical Medicine, School of Medicine, National Yang-Ming University, Taipei 11221, Taiwan; 7 Department of Neurology, Neurological Institute, Taichung Veterans General Hospital, Taichung 40705, Taiwan; 8 Division of Allergy, Immunology and Rheumatology, Department of Internal Medicine, Taichung Veterans General Hospital, Taichung 40705, Taiwan; 9 Department of Family Medicine, Taichung Veterans General Hospital, Taichung 40705, Taiwan; 10 Division of Endocrinology and Metabolism, Department of Internal Medicine, Taichung Veterans General Hospital, Taichung 40705, Taiwan

**Keywords:** aged, geriatric assessment, length of stay, readmission, quality of life

## Abstract

We evaluated the predictability of self-reported Health-related quality of life (HRQoL) assessed by the 3-level 5-dimensional Euro-Quality of Life tool (EQ-5D-3L) and the EQ-Visual Analog Scale (EQ-VAS) on clinical outcomes of elderly patients who were admitted to an acute geriatric ward. A total of 102 participants (56.9% men) with a median age of 81.0 years (interquartile range or IQR: 76.0–85.3 years) were studied. The age-adjusted Charlson comorbidity index was 5.0 (IQR: 4.0–6.0) with a median length of stay (LOS) of 9.0 days (IQR: 7.0–15.0 days). No death occurred during hospitalization, and within 30 days after discharge, 15 patients were readmitted. During hospitalization, the EQ-5D-3L index was 0.440 at admission and that improved to 0.648 at discharge (*p* < 0.001). EQ-VAS scores also improved similarly from 60 to 70 (*p* < 0.001). Physical, cognitive function, frailty parameters (hand grip strength and walking speed), and nutritional status at admission all improved significantly during hospitalization and were related to EQ-5D-3L index or EQ-VAS scores at discharge. After controlling for relevant factors, EQ-5D-3L index at admission was found to be associated with LOS. In addition, EQ-VAS was marginally related to readmission. HRQoL assessment during hospitalization could be useful to guide clinical practice and to improve outcome.

## 1. Introduction

Population ageing in most countries increases the demand on social and health-care services, and hospital admission is one indicator of the increased demand [1,2]. The course of admission and hospitalization are major stresses for the vulnerable elderly people and results in a loss of independent activities of daily life (ADL) [3]. Worse, many of them experience repeated admissions. Rates of early readmission (within 30 days after discharge) are reported to vary between 11% to 19% depending on care settings and population groups [4]. High readmission rates are associated with poorer patient outcomes, incurring substantial economic burden [5]. It has been proposed that sociodemographics, comorbidities, functional status, and previous health-care utilizations to a great extent influence readmission rates [6].

Quality of life (QoL) is a multidimensional term, consisting of several aspects of life. When used in reference to health care, QoL means health-related quality of life (HRQoL) and can be defined as a functional effect on medical conditions and/or its treatment upon a patient’s physical, social, and emotional well-being [7]. In healthy and chronically ill community-dwelling elderly people, higher HRQoL is associated with longer survival and lower admission rates [8,9,10,11,12,13,14]. For the acutely hospitalized patients, HRQoL at admission predicts subsequent risk of mortality and functional decline [9]. HRQoL is also dynamic, and it could change during hospitalization [15]. HRQoL during hospital stay can provide an information profile of a patient for health-care providers and can assist treatment decisions during hospitalization.

In 2018, Taiwan was an aged country with the percentage of citizens over 65 years of age at higher than 14% [16]. With the advancing ageing population, geriatric wards have been established in several tertiary medical centers over the entire island and have provided services based on principles of comprehensive geriatric assessment and team management, which included physical activity, nutrition, cognition, mood, frailty, life quality, etc. [17]. However, studies on HRQoL in acutely sick patients are scarce in Taiwan. No study has yet been done regarding the relationship between early HRQoL and hospital outcomes, like length of stay (LOS), discharge or death, and readmission. Based upon the effects of HRQoL on patient’s physical, social, and emotional well-being assessment, we hypothesized that HRQoL can be used as a predictor of clinical outcome. To test the hypothesis, the study aimed first to examine the relationship of HRQoL and relevant factors with LOS and readmission within 30 days post-discharge in ≥65 years old patients admitted to a geriatric ward. We also investigated possible changes in HRQoL during hospitalization and determined the influencing factors.

## 2. Materials and Methods

### 2.1. Participants

The study was conducted at the geriatric ward in a medical center in Taiwan from 1 March to 31 October 2018. The participants enrollment criteria included those who were aged ≥65 years and were admitted due to acute illness with geriatric syndromes, i.e., development of physical dependency within weeks prior to admission because of acute illness. Patients were excluded if they were completely physically dependent before admission, had terminal illness with an expected survival time of less than 6 months, or had limited ability to receive comprehensive geriatric assessment. At admission, patients’ general demographic data were recorded in medical history by the physicians, including age, gender, education, and marital and religious status. Besides, we assessed patients’ medical histories and comorbid conditions and recorded their diagnosed diseases, medications, and their age-adjusted Charlson Comorbidity Index (ACCI) [18]. In this study, we consecutively enrolled 102 participants whose baseline data were presented in Table 1. Their median age was 81.0 years (interquartile range (IQR): 76.0–85.3 years) with 56.9% men (Table 1). The majority (99%) of them were married, with 37.3% widows or widowers and 2.0% divorced. The participants’ education levels were 20.6% illiterate, 5.9% barely literate, 33.3% primary school, 28.4% high school, and 11.8% university. Regarding religious belief, 80.4% had a religion, 54.9% were folk believers, 9.8% practiced Buddhism, 9.8% were Protestant or Catholics, and 5.9% practiced Taoism. The top three leading causes of admission were pneumoniae (40.2%), urinary tract infection (26.5%), and cellulitis (15.7%). The most common comorbidities were hypertension, diabetes, and chronic obstructive pulmonary disease. Polypharmacy was found with 77 participants, with a median ACCI score of 5 (IQR: 4.0–6.0 score). LOS was 9.0 days (IQR: 7.0–15.0 days), and 15 participants had readmission but with no death during the study period. In the geriatric ward, the participants received coordinated care of the multidisciplinary team, including geriatricians, nurses, physiotherapists, dietitians, pharmacists, etc. For example, rehabilitation plans in addition to treatment of acute illness were implemented by physicians, physiotherapists, and nurses to ensure mobilization during hospitalization. The study was reviewed and approved by the Institutional Review Board of the medical center (IRB no: CE18141B).

### 2.2. Assessment Procedures

At the times of both admission and discharge, life quality was assessed with the EQ-5D by well-trained nurses. The three-level five-dimensional Euro-Quality of Life tool (EQ-5D-3L) comprises the following: mobility, self-care, usual activities, pain/discomfort, and anxiety/depression. Each dimension was assessed in terms of three levels: no problems, moderate problems, and severe problems. Data were converted to indices calculated using the anchor Taiwan population weights [19]. The nominal range of EQ-5D-3L index was between 0 to 1, with 0 equivalent to death and 1 equivalent to perfect health. Negative values represented health statuses worse than death. The EQ-visual analogue scale (EQ-VAS), part of EQ-5D, was a self-rated health index scaled between 0 and 100, with high scores representing good health status. Besides, the nurses also conducted comprehensive geriatric assessments (CGAs), including the Mini-Mental State Examination (MMSE), 5-item Geriatric Depression Scale (GDS-5), Activities of Daily Living (ADL), Instrumental Activities of Daily Living (IADL), Mini-Nutritional Assessment (MNA), Hand Grip Strength (HGS), Walking Speed (WS), and Timed Up and Go test (TUG) [17]. The cognitive status was measured with the MMSE, which had scores ranging from 0 to 30, with high scores indicating good cognitive ability. The mood condition was screened by GDS-5, with total scores ranging from 0 to 5, with 2 or higher indicating risks for depression. The physical function was reflected by the Barthel index of ADL and the scores of the Lawton IADL scale. Nutrition status was assessed by MNA scoring between 0 and 30, with >23.5 indicating normal nutritional status. Mobility was assessed using HGS, WS, and TUG. Using a handheld dynamometer (Smedley’s Dynamometer, TTM, Tokyo, Japan), HGS was measured, and the best measurement of three times was recorded on the dominant hand with the subject in a sitting posture and elbow flexed at 90°. We also recorded the 6-m WS. According to the definition of sarcopenia by the Asia Working Group for Sarcopenia in 2014, low HGS was defined as HGS <26 kg for men and <18 kg for women. Low WS was defined as <0.8 m/s. TUG was used to measure various components like coordination, balance, and burst lower extremity strength. The overall outcomes, including the LOS, readmission within 30 days, and mortality were analyzed. At one month and three months after discharge, we contacted the patient or a relative by telephone and the electronic patient file was checked to determine participant’s discharge destination.

### 2.3. Statistical Analyses

Continuous variables were expressed as median and interquartile range (IQR, 25%−75%). Categorical data were expressed as number and percentage of the total number of participants. Paired comparisons were made using the Wilcoxon signed rank test or Friedman test for continuous variables and McNemar’s or Cochran’s Q test for categorical variables. The univariate and multivariate regression analyses were used to assess relationships between either EQ-5D, LOS, or readmission and the demographic and clinical data. In the multivariate analysis, variables with *p* values less than 0.05 from univariate analysis were included as cofounders in an adjustment. Continuous variables with skewed distributions were normalized by logarithmic transforms. As negative values of the EQ-5D-3L index were considered having a health status worse than death, we included for analysis only 0 and positive values. Statistical analyses were performed using SPSS version 22.0 (SPSS Inc., Chicago, IL, USA). Statistical significance was set at *p* < 0.05.

## 3. Results

In all 102 participants, the EQ-5D-3L index, EQ-VAS, MMSE, ADL scores, TUG, and WS all improved significantly in comparison with those at admission (Table 2). The EQ-5D-3L index at admission was 0.440 and improved to 0.648 at discharge, and the EQ-VAS score was similarly improved from 60 to 70 (both *p* < 0.001). EQ-5D-3L index at admission was associated with GDS-5, ADL, IADL, MNA, and HGS (data not shown). Physical and cognitive functions, frailty parameters (HG and WS), and nutritional status were all related to EQ-5D-3L index and EQ-VAS scores at discharge using the univariate regression analysis (Table 3 and Table 4). However, patients’ marital status, education levels, and religions were not associated with EQ-5D-3L index and EQ-VAS scores either at admission or discharge. After adjustment for the other covariates, multivariate regression analysis showed that only ADL (*p* < 0.001) and EQ-5D-3L index at admission (*p* = 0.002) were independently positively correlated with EQ-5D-3L index at discharge and that only EQ-VAS (*p* < 0.001) and HGS (*p* = 0.029) at admission were independently positively correlated EQ-VAS scores at discharge.

To determine the relevant factors of LOS, we found that EQ-5D-3L index at admission, GDS-5, ADL, MNA, TUG, and WS were all related to LOS in the univariate regression analysis and that, after adjustment of the other variables, the EQ-5D-3L was the single index remaining associated with LOS at significant levels (Table 5). For readmission, we found marginal associations between age, gender, HGS or EQ-VAS score, and readmission in the univariate logistic regression analysis. After controlling the other covariates, only age remained correlated with readmission (Table 6).

## 4. Discussion

The principal finding of this study is that HRQoL, as measured with the EQ-5D instrument, improved significantly in a group of elderly patients admitted to our acute geriatric ward. Also, HRQoL at admission was associated with both LOS and readmission. Several previous studies have reported that HRQoL returns to population norms one year or longer after discharge from intensive care units [20]. In our study, we found that HRQoL showed partial improvements even as early as at discharge. Such partial recovery could be the results of functional readaptation by physical and psychological rehabilitations and other medical services during hospitalization.

For elderly patients hospitalized for acute illness, HRQoL is known to be an important determinant of hospital outcomes (e.g., LOS) [9,20,21,22,23], although in dispute, other studies reported no relationship [24]. Higher baseline HRQoL is considered to have better functional reserves or ability to recover and could thus predict shorter hospital stays [10]. This method of patient-reported outcomes can be used for clinical monitoring—allowing changes in clinical condition of the patients being detected during hospitalization.

Readmission is a rather complex phenomenon and is often difficult to predict. Previous studies reported that a frail state or low functional status is associated with readmission [25,26]. In some cases, poorer HRQoL is associated with a greater chance of death [9,11,12,13]. In our present study, EQ-VAS scores were only marginally related to readmission. This was in line with a previous study which showed that HRQoL is an independent predictor of readmission [27]. The reason why the EQ-5D index score was not associated with readmission was not clear. It could be that EQ-VAS scores and EQ-5D indices are not identical parameters, since the EQ index reflects health status based on preferences of the general population and the EQ-VAS score reflects personal health status. Nevertheless, information derived from self-reported health status, HRQoL, anxiety, depression, and symptom burden can help identify those patients at risk of future readmission. It is likely that, if medical staff were to provide the person-centered approach, patient’s expectations, worries, thoughts, and resources can be more readily identified and support to patients in their self-care at discharge can be better established [8].

The host of geriatric syndromes include polypharmacy, cognitive and functional impairment, visual and hearing loss, falling, malnutrition, sarcopenia and frailty, and urinary incontinence. These conditions are highly prevalent in older people and are often associated with multiple comorbidities, likely contributing to lower scores in HRQoL [28]. However, nutritional and rehabilitation programs given to elderly people can improve their HRQoL [29,30]. In this study, 69.6% of participants had malnutrition or malnutrition risk and 71.3% had problems with mobility, self-care, or usual activities. These conditions were associated with lower EQ-5D-3L indices and EQ-VAS scores. With identifying geriatric problems by using CGA, multidisciplinary services in addition to illness treatment were provided, including pharmacist recommendations for polypharmacy, dietitian-offered dietary advice for malnutrition, and physiotherapist/occupational therapist-provided personalized rehabilitation programs, etc. Afterwards, physical function, frailty parameters (HGS and WS), as well as nutrition of the study subjects were all improved at discharge in association with improved EQ-5D-3L indices and EQ-VAS scores, findings which were in line with the previous literature. Given the association between HRQoL, as assessed by EQ-5D; LOS; and readmission, our findings had a clinical implication of initiating interventions early to improve outcome.

There are some limitations of this study. First, the sample size was limited and follow-up time was short. Thus, our results cannot be generalized to other hospitals. Second, there was no control group. It remains unclear to what extent the improved HRQoL observed at discharge reflected spontaneous recovery to baseline conditions. Third, no evaluation was done between EQ-5D indices or EQ-VAS scores and biochemical factors, medications, and the other interventional procedures, which are also determinants of discharge outcome. A larger scale and longer-duration prospective study in the future can determine better the predictive value of HRQoL on the outcome of such elderly patients.

## 5. Conclusions

In acutely admitted elderly patients, HRQoL improved in association with geriatric conditions. Better baseline physical, nutritional, and frailty conditions were all associated with better HRQoL at discharge. HRQoL at admission was correlated with risks of prolonged hospital stay and readmission. We proposed that it is important to evaluate patient-perceived health-related life quality to guide clinical practice and intervention during hospitalization.

## Figures and Tables

**Table 1:** Characteristics of the patients (*n* = 102).

Characteristics	Median	(IQR)
**Age (years)**	81	(76–85.3)
**Gender, *n* (%)**		
Male	58	(56.9%)
Female	44	(43.1%)
**Marital Status, *n* (%)**		
Single	1	(1.0%)
Married	61	(59.8%)
Widowed	38	(37.3%)
Divorced	2	(2.0%)
**Educational level, *n* (%)**		
Illiterate	21	(20.6%)
Literate	6	(5.9%)
Primary school	34	(33.3%)
Junior high school	11	(10.8%)
Senior high school	18	(17.7%)
University	12	(11.8%)
**Religion, *n* (%)**		
None	20	(19.6%)
Buddhist	10	(9.8%)
Taoist	6	(5.9%)
Christian	8	(7.8%)
Catholics	2	(2.0%)
Folk beliefs	56	(54.9%)
**Polypharmacy, *n* (%)**		
No	25	(24.5%)
Yes	77	(75.5%)
**Age-adjusted Charlson comorbidity Index (ACCI)**	5	(4–6)
**Length of stay (days)**	9	(7–15)
**Readmission, *n* (%)**		
No	87	(85.3%)
Yes	15	(14.7%)

Continuous data were expressed median and interquartile range (IQR); categorical data were expressed as a number and percentage.

**Table 2:** Changes of the 3-level 5-dimensional Euro-Quality of Life tool (EQ-5D-3L) index, EQ-Visual Analog Scale (EQ-VAS), and comprehensive geriatric assessment parameters between admission and discharge.

Dimension	Measures	Item Number	Score Range	Admission	Discharge	*p* Value
				Median (IQR)	Median (IQR)	
**Life quality**	3-level version of European Quality of life-5 dimensions index (EQ-5D-3L index)	5	−0.623–1	0.440 (0.165–0.924)	0.648 (0.371–1)	<0.001
	EQ visual analogue scale (EQ-VAS)	1	0–100	60 (50–80)	70 (60–85)	<0.001
**Cognition**	Mini-mental state examination (MMSE)	11	0–30	24 (20–28)	25 (21–29)	<0.001
**Mood**	5-item Geriatric Depression Scale (GDS-5)	5	0–5	1 (0–2)	1 (0–2)	0.334
**Functional status**	Barthel index (ADL)	10	0–100	55 (35–75)	70 (43.8–85)	<0.001
	Lawton scale (IADL)	8	0–8	3 (1–4)	3 (1–4)	0.109
**Nutrition**	Mini-Nutritional Assessment (MNA)	18	0–30	21 (18–24)	21 (18.4–24)	0.465
**Frailty**	Hand Grip Strength (HGS)	-	Kilogram	17.1 (12.8–24.1)	Not examined	-
	Walking Speed (WS)	-	Meter/second	0.52 (0.4–0.8)	0.58 (0.4–0.8)	<0.001
	Timed Up and Go test (TUG)	-	Seconds	15.7 (13.9–19.8)	14.6 (12.7–18.2)	0.001

Continuous data were expressed median and interquartile range (IQR).

**Table 3:** Discharge EQ-5D-3L index (Log_10_) with comprehensive geriatric assessment parameters at admission.

	Univariate Linear Regression		
	B	Beta (β)	*p* Value
EQ-5D-3L index	0.54	0.82	<0.001
EQ-VAS	0.00	0.18	0.114
Age	−0.01	−0.16	0.156
**Gender**			
Male	ref.		
Female	−0.01	−0.02	0.834
**Educational level**			
Illiterate	−0.17	−0.28	0.021
Literate	−0.13	−0.12	0.275
Primary school	ref.		
Junior high school	−0.09	−0.12	0.307
Senior high school	−0.24	−0.37	0.002
University	0.06	0.09	0.456
**Marital Status**			
Single/Widowed/Divorced	ref.		
Married	−0.05	−0.10	0.361
**Religion**			
None	ref.		
Yes	−0.01	−0.02	0.892
**CGA at admission**			
ACCI	−0.02	−0.10	0.366
MMSE	0.01	0.25	0.023
GDS-5	−0.10	−0.58	<0.001
ADL	0.01	0.61	<0.001
IADL	0.06	0.52	<0.001
MNA	0.03	0.53	<0.001
TUG	−0.01	−0.34	0.031
WS	0.25	0.41	0.004
HGS	0.01	0.37	0.001

ref., reference; B, unstandardized coefficients; Beta, standardized coefficients.

**Table 4:** Discharge EQ-VAS (Log_10_) with comprehensive geriatric assessment parameters at admission.

	Univariate Linear Regression		
	B	Beta (β)	*p* Value
EQ-5D-3L index	0.11	0.36	0.001
EQ-VAS	0.01	0.78	<0.001
Age	0.00	0.02	0.884
**Gender**			
Male	ref.		
Female	−0.06	−0.23	0.030
**Educational level**			
Illiterate	−0.01	−0.02	0.883
Literate	−0.07	−0.12	0.287
Primary school	ref.		
Junior high school	0.02	0.06	0.609
Senior high school	−0.002	−0.01	0.958
University	0.04	0.09	0.429
**Marital Status**			
Single/Widowed/Divorced	ref.		
Married	0.04	0.14	0.194
**Religion**			
None	ref.		
Yes	0.002	0.01	0.955
**CGA at admission**			
ACCI	0.00	−0.03	0.789
MMSE	0.00	0.12	0.270
GDS-5	−0.02	−0.20	0.062
ADL	0.00	0.28	0.008
IADL	0.01	0.12	0.258
MNA	0.01	0.36	0.001
TUG	0.00	−0.35	0.028
WS	0.06	0.18	0.221
HGS	0.01	0.36	0.001

ref., reference; B, unstandardized coefficients; Beta, standardized coefficients.

**Table 5:** Length of stay (Log10) with the EQ-5D-3L index, EQ-VAS, and comprehensive geriatric assessment parameters at admission.

	Univariate Linear Regression		
	B	Beta (β)	*p* Value
EQ-5D-3L index	−0.26	−0.37	<0.001
EQ-VAS	0.00	−0.15	0.152
Age	0.00	0.00	0.977
**Gender**			
Male	ref.		
Female	0.06	0.10	0.330
**Educational level**			
Illiterate	0.09	0.11	0.313
Literate	0.18	0.14	0.192
Primary school	ref.		
Junior high school	−0.04	−0.04	0.708
Senior high school	0.12	0.15	0.186
University	0.01	0.01	0.912
**Marital Status**			
Single/Widowed/Divorced	ref.		
Married	−0.07	−0.11	0.288
**Religion**			
None	ref.		
Yes	0.00	−0.01	0.955
**CGA at admission**			
ACCI	−0.01	−0.06	0.537
MMSE	−0.01	−0.10	0.317
GDS-5	0.04	0.21	0.032
ADL	0.00	−0.23	0.019
IADL	−0.03	−0.17	0.090
MNA	−0.02	−0.23	0.020
TUG	0.01	0.33	0.031
WS	−0.45	−0.37	0.009
HGS	−0.01	−0.18	0.078

ref., reference; B, unstandardized coefficients; Beta, standardized coefficients.

**Table 6:** Readmission with EQ-5D-3L index, EQ-VAS, and comprehensive geriatric assessment parameters at admission.

	Univariate Model		
	OR	(95% CI)	*p* Value
EQ-5D-3L index	1.32	(0.37–4.72)	0.667
EQ-VAS	1.03	(1.00–1.06)	0.085
Age	1.20	(1.06–1.35)	0.004
Gender (F vs. M)	0.28	(0.07–1.06)	0.062
**Educational level**			
Illiterate	ref.		
Literate	10.00	(0.72–138.68)	0.086
Primary school	2.67	(0.28–25.64)	0.396
Junior high school	2.00	(0.11–35.41)	0.636
Senior high school	5.71	(0.58–56.73)	0.137
University	6.67	(0.61–73.19)	0.121
* Marital Status (Y vs. N)	1.36	(0.45–4.11)	0.581
Religion (Y vs. N)	0.97	(0.25–3.83)	0.967
Polypharmacy (Y vs. N)	2.34	(0.49–11.15)	0.287
**CGA at admission**			
ACCI	1.35	(0.93–1.97)	0.115
MMSE	1.05	(0.94–1.18)	0.391
GDS-5	0.92	(0.63–1.35)	0.677
ADL	1.00	(0.97–1.02)	0.930
IADL	0.98	(0.74–1.30)	0.886
MNA	1.01	(0.88–1.15)	0.936
TUG	0.98	(0.88–1.10)	0.750
WS	0.12	(0.00–3.74)	0.229
HGS	1.07	(1.00–1.15)	0.056
Length of stay	0.99	(0.95–1.04)	0.814

* Marital status: Y meant married and N meant single, divorced, or widowed. OR, odds ratio; CI, confidence interval.

## Abbreviations

ACCI: age-adjusted Charlson Comorbidity Index; ADL: activities of daily living; CGA: comprehensive geriatric assessment; EQ-5D-3L: 3-level version of European Quality of life-5 dimensions; EQ-VAS: EQ visual analogue scale; GDS-5: five-item Geriatric Depression Scale; HGS: Hand Grip Strength; HRQoL: Health-related quality of life; IADL: Instrumental Activities of Daily Living; IQR: interquartile range; LOS: length of stay; MMSE: mini-mental state examination; MNA: Mini-Nutritional Assessment; TUG: Timed Up and Go test; WS: Walking Speed.
